# Perspectives of infectious disease specialists on shared decision-making for fever of unknown origin: a national survey in China

**DOI:** 10.3389/fpubh.2026.1779890

**Published:** 2026-07-23

**Authors:** Ling Qin, Zhiyi Pei, Xiaofeng Kang, Taisheng Li, Ying Ge

**Affiliations:** 1 Department of Infectious Diseases, Peking Union Medical College Hospital, Chinese Academy of Medical Sciences & Peking Union Medical College, Beijing, China; 2 School of Nursing, Chinese Academy of Medical Sciences & Peking Union Medical College, Beijing, China; 3 State Key Laboratory of Complex, Severe and Rare Diseases, Peking Union Medical College Hospital, Chinese Academy of Medical Sciences & Peking Union Medical College, Beijing, China

**Keywords:** China, fever of unknown origin, infectious disease specialists, national survey, shared decision-making

## Abstract

**Background:**

This study aimed to investigate the attitudes of infectious disease specialists toward shared decision-making (SDM) and identify the perceived barriers to its implementation in the management of fever of unknown origin (FUO) in China.

**Methods:**

A nationwide, cross-sectional, online survey was conducted in July 2025 among infectious disease specialists at Chinese general hospitals. Eligible participants were infectious disease specialists registered and working in Chinese general hospitals. Exclusion criteria were: (1) individuals without prior experience treating patients with FUO; (2) individuals practicing in other specialties; (3) individuals unable to complete the survey on digital devices; and (4) questionnaires completed in under 120 s. The 27-item questionnaire assessed: (1) attitudes and implementation tendencies regarding the six steps of SDM; (2) perceived barriers to SDM; and (3) perceived effectiveness using the 9-item Shared Decision-Making Questionnaire-Doctor version (SDM-Q-Doc). Data were analyzed using descriptive statistics, univariate tests, Random Forest model, Least Absolute Shrinkage and Selection Operator (LASSO) regression, and multivariate linear regression.

**Results:**

Of the 429 responses received, 386 were eligible (67.4% females and 32.6% males). The mean age of participants was 41.42 years. Only 107 participants (27.7%) reported prior knowledge of SDM, and 33 (8.5%) had received formal training. Mean scores were 4.46/6 for attitudes, 3.52/9 for barriers, and 40.77/45 for perceived effectiveness. Higher perceived effectiveness scores were significantly associated with gender, managing≥10 FUO cases annually, frequent participation in multidisciplinary discussions, prior knowledge of SDM, and positive attitudes toward SDM. Primary barriers included unequal information between physicians and patients (*n* = 256, 66.3%), patients’ limited knowledge (*n* = 243, 63.0%), and insufficient technical support and training (*n* = 242, 62.7%). Multivariate analysis identified “presenting options clearly,” “inviting patients to participate in SDM,” “facilitating deliberation and decision-making” and “gender” as independent predictors of perceived effectiveness.

**Conclusion:**

Infectious disease specialists in China demonstrated positive attitudes toward SDM in FUO management, which correlate with higher perceived effectiveness. To promote consistent and high-quality SDM in complex diagnostic scenarios, interventions should focus on structured training programs and the integration of decision-support tools into continuing medical education.

## 1 Introduction

Fever of unknown origin (FUO) continues to pose a significant diagnostic challenge worldwide (1). Defined as a prolonged fever without an identifiable source despite a thorough evaluation, FUO affects a substantial proportion of adult patients globally. FUO evaluation always begins with a comprehensive medical history, physical examination, and initial diagnostic investigations. While physicians should energetically pursue the final diagnosis (2), the etiology remains unknown in 10–50% of FUO patients, and this figure is increasing (3–5). Moreover, the diagnostic process for FUO is often lengthy and resource-intensive, with over 200 potential causes ranging from infections and malignancies to autoimmune diseases and other conditions (6). For many patients, the prolonged uncertainty itself becomes a source of anxiety, frustration, and reduced quality of life (7). Physicians also face considerable cognitive and emotional stress as they navigate uncertain diagnostic pathways and balance thorough investigation with patient expectations and safety.

In such complex diagnostic landscapes, shared decision-making (SDM)—a collaborative process in which physicians and patients make health decisions together based on clinical evidence and patient preferences—has emerged as a valuable tool to enhance trust, communication, and patient-centered care (8). Originally conceptualized in the context of preference-sensitive decisions in chronic diseases such as cancer, cardiovascular disease, and mental health, SDM has since been widely endorsed across diverse clinical settings (9–11). In pediatric care, particularly when managing acute febrile illnesses in children, SDM has shown promise in reducing unnecessary antibiotic use and improving parental satisfaction (12). However, existing SDM models predominantly operate under the premise of a confirmed diagnosis, focusing primarily on treatment selection, such as drug selection in cardiovascular diseases or surgical options in cancer. In contrast, adult FUO presents a unique challenge: SDM must address prolonged diagnostic ambiguity (13), weigh the risks and benefits of increasingly invasive or expensive investigations, and manage patient anxiety across a broad differential diagnosis (14, 15). Despite the critical need for collaborative uncertainty management, the application of SDM in adult FUO remains strikingly unexplored.

Unlike Western healthcare systems, where SDM is deeply rooted in individual autonomy and supported by longer consultations, implementation in China faces unique systemic barriers (16). Chinese general hospitals operate under extreme patient volumes and severe time constraints, with infectious disease departments managing the majority of adult FUO cases. Within this high-pressure environment, physicians must navigate clinical ambiguity while balancing thorough investigation with patient expectations. Consequently, structured SDM may facilitate transparent communication and ethically sound care in complex diagnostic scenarios.

While previous studies suggest that communication improves medical decisions and patient experience in complex conditions (17, 18), empirical data regarding SDM in the highly uncertain and culturally distinct context of FUO are scarce. In light of the limited research, several key questions remain unanswered. What are the attitudes of infectious disease physicians toward SDM in FUO cases? What barriers are perceived as hindering the implementation of SDM in this clinical setting? Which factors predict the integration of SDM behaviors into real-world clinical practice? To address these questions, we conducted a nationwide cross-sectional survey of infectious disease specialists in China. The aim was to characterize their current practices, perceptions and challenges regarding SDM in the management of adult FUO.

## 2 Methods

### 2.1 Sample and data collection

This nationwide cross-sectional study was conducted in July 2025. Data were collected via an online survey, in accordance with the Checklist for Reporting Results of Internet E-Surveys (CHERRIES) guidelines (19). The inclusion criterion was infectious disease specialists who were registered and working in Chinese general hospitals. Exclusion criteria included: (1) individuals without prior experience of treating patients with FUO; (2) individuals practicing in other specialties; (3) individuals unable to complete the survey on digital devices; and (4) questionnaires completed in under 120 s. Informed consent was obtained from all participants. Their personal information was anonymized and kept confidential and was used exclusively for research purposes. The survey was administered via the WeChat mobile app using the Questionnaire Star platform (www.wjx.cn) and employed convenience sampling to recruit infectious disease specialists nationwide. Accessible only via WeChat, the questionnaire permitted a single submission per unique account. It comprised mandatory closed questions with predefined response options, requiring complete responses before submission. Participation was voluntary, and respondents were encouraged to answer truthfully based on their actual experiences.

### 2.2 Survey items

**Figure 1:**
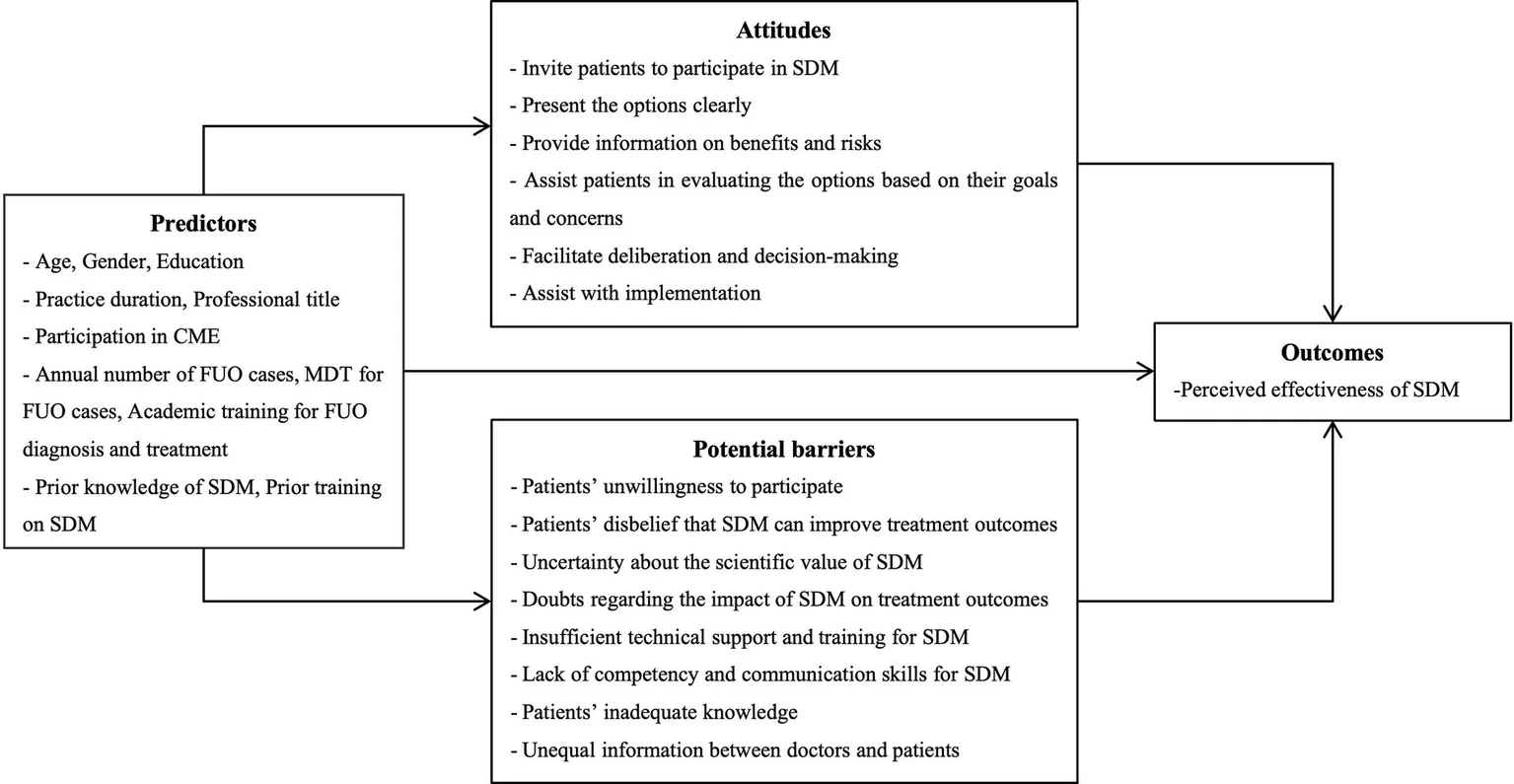
Conceptual framework of factors influencing infectious disease specialists’ perceived effectiveness of SDM in FUO management. This model illustrates the theoretical relationships between the predictor variables, attitudes regarding the six steps of SDM, potential barriers, and the outcome variable.

The 27-item structured questionnaire (Table 1) was adapted from the framework of Yang et al. (20). The conceptual relationship between the measured domains is illustrated in Figure 1. The instrument assessed the following domains: (1) Attitudes and implementation tendencies regarding the six steps of SDM in daily practice (Table 1, Questions 12–17) (21): ① Inviting patients to participate in SDM; ② Presenting the options clearly; ③ Providing information on benefits and risks; ④ Helping the patients evaluate the options based on their goals and concerns; ⑤ Facilitating deliberation and decision-making; ⑥ Assisting with implementation. Each item was answered with “yes” (score as 1) or “no” (score as 0). The total score ranges from 0 to 6, with a higher score indicating a more favorable attitude toward and tendency to implement SDM. (2) Potential barriers to SDM implementation (Table 1, Questions 18): Participants were asked to rate a list of potential barriers identified in prior systematic reviews published in English and French (22) and Chinese (23). Each item was answered with “yes” (scored as 1) or “no” (scored as 0). The total score ranges from 0 to 9, with higher scores indicating greater perceived barriers to SDM implementation. (3) Perceived effectiveness of SDM implementation strategies (Table 1, Questions 19–27): The nine-item Shared Decision-Making Questionnaire-Doctor version (SDM-Q-Doc) was used to assess a clinical scenario involving bone marrow biopsy for FUO. This instrument has been shown to be highly acceptable, feasible and reliable (24), measuring the quality of the medical decision-making process from the physician’s perspective. Scoring is conducted using a Likert-type scale, where 0 represents “complete disagreement” and 5 represents “complete agreement.” The total score ranges from 0 to 45, with higher scores indicating greater implementation of SDM in clinical practice. The reliability of the adapted SDM-Q-Doc was assessed using Cronbach’s alpha. In this study, the instrument demonstrated high internal consistency, with a Cronbach’s alpha coefficient of 0.919.

**Table 1:** The 27-item questionnaire on the national survey.

Item	Question
1	What is your age?
2	What is your gender?
3	What is your highest degree?
4	How long have you been working in the field of infectious diseases?
5	What is your current professional title?
6	Have you ever participated in continuing medical education (CME)?
7	How many cases of fever of unknown origin (FUO) do you treat per year?
8	How many times do you participate in multidisciplinary discussions for FUO diagnosis and treatment per year?
9	How many times do you receive online/offline academic training for FUO diagnosis and treatment per year?
10	Have you ever known about shared decision-making (SDM)?
11	Have you ever received training on SDM?
12	Do you involve patients in medical decision-making during the diagnosis and treatment of FUO?
13	Do you provide detailed information about the available treatment options for patients with FUO?
14	Would you ask a patient, ‘Different people have different priorities. Considering the potential benefits and risks of these options, which outcomes are you expect to achieve? Which outcomes are you concerned about?’
15	After clarifying a patient’s treatment preferences, would you help them weigh the potential benefits and risks of the available options?
16	During the diagnostic and treatment process, would you say to a patient, “Let us talk about what the next steps are”?
17	Do you use doctor–patient collaborative decision-making network resources in your daily practice?
18	What do you think are the barriers to achieving SDM with patients?
For Question 19 to 27, a real case of FUO requiring evaluation for hematologic diseases is described. The patient was unwilling to continue treatment due to concerns about the potential adverse effects of a bone marrow biopsy, including pain, bleeding, and infection. You have already discussed the next steps in the treatment plan with her. Please answer the following questions. A score of 0 indicates complete disagreement, and a score of 5 indicates complete agreement (based on the SMD-Q-Doc questionnaire).	
19	I clearly explained to the patient that the bone marrow biopsy is critically important, and that the decision to continue treatment must be made by the patient.
20	I hope to clearly understand how the patient made the decision to refuse the bone marrow biopsy.
21	I have informed the patient of the different treatment options available based on the current clinical situation.
22	I clearly explained the advantages and disadvantages of the different treatment options to patients.
23	I helped the patient understand all the relevant information about the disease itself and the bone marrow biopsy procedure.
24	I asked the patient which treatment option they would prefer.
25	I discussed with the patient and carefully weighed the pros and cons of not performing a bone marrow puncture.
26	We jointly determined the next treatment plan.
27	I reached an agreement with the patient and decided whether to perform a bone marrow puncture biopsy, as well as the methods and details of this medical treatment.

### 2.3 Data analysis

Continuous variables were reported as mean (Standard Deviation, SD) and compared using independent t-tests or one-way analysis of variance (ANOVA). Categorical variables were presented as frequencies and percentages (*n*, %), and analyzed using chi-square test.

To ensure both robust predictor identification and clinical interpretability, a three-stage analytical framework was employed. First, variables demonstrating statistical significance in univariate analyses were entered into a Random Forest model. As a non-parametric machine learning approach, Random Forest was utilized to rank variables by their percentage increase in mean squared error (%IncMSE), with higher %IncMSE values indicating a greater explanatory contribution of the variable. This allowed for the capture of complex non-linear relationships and feature interactions while remaining unaffected by multicollinearity. Second, Least Absolute Shrinkage and Selection Operator (LASSO) regression was performed for dimensionality reduction. By applying a penalty term to shrink less informative coefficients to zero, the LASSO model effectively mitigated over-fitting and addressed multi-collinearity inherent in high-dimensional survey data. This was done using the glmnet function in RStudio. Finally, the variables identified by LASSO were included in a multivariable linear regression model. This hybrid approach leverages the predictive strengths of machine learning for feature selection while preserving the formal clinical interpretability of traditional regression. Statistical significance was defined as a two-sided *p*-value < 0.05. SPSS version 27.0 (IBM Corp, Armonk, New York) and RStudio version 4.4.1 (Posit, PBC, Boston, Massachusetts) were used for data analysis.

## 3 Results

### 3.1 Participant demographics

Of the 429 questionnaires returned, 43 were excluded after screening: 10 contained logical inconsistencies, 29 came from staff at infectious disease hospitals, and 4 were from physicians with no experience of managing patients with FUO. The final sample comprised 386 participants from 23 provinces. The overall mean (SD) age of all participants was 41.42 (8.01) years. Of these, 260 (67.4%) were female with a mean (SD) age of 40.29 (7.81) years and 126 (32.6%) were male with a mean (SD) age of 43.76 (7.94) years. Almost half (*n* = 182, 47.2%) held a master’s degree, and 50 (13%) had a doctoral degree. In terms of professional titles, 126 participants (32.6%) were associate professors, and 74 (19%) were full professors. Over one-third (*n* = 120, 31.1%) had worked in infection disease departments for more than 15 years, and 223 (57.8%) had received continuing medical education (CME). The majority of participants (*n* = 316, 81.9%) managed over 10 FUO cases annually, and 164 (42.5%) participated in over 10 multidisciplinary team (MDT) discussions for FUO each year. Only 107 participants (27.7%) knew about SDM, while 279 (72.3%) reported no prior knowledge of SDM, and 33 (8.5%) had received SDM training. Table 2 provides a summary of the participants’ additional characteristics.

**Table 2:** Characteristics of the participants.

Variables	*N* = 386	%	Attitude^a^		Potential barrier^b^		Perceived effectiveness^c^	
			Mean (SD)	*P*	Mean (SD)	*P*	Mean (SD)	*P*
Total			4.46 (1.38)		3.52 (1.76)		40.77 (6.3)	
Age (years)								
< 35	82	21.2	4.65 (1.39)	0.16	3.82 (2.01)	0.083	40.45 (5.78)	0.607
≥ 35	304	78.8	4.40 (1.38)		3.44 (1.68)		40.86 (6.44)	
Gender								
Male	126	32.6	4.38 (1.49)	0.459	3.65 (1.90)	0.302	39.51 (6.89)	0.009
Female	260	67.4	4.49 (1.33)		3.45 (1.68)		41.38 (5.91)	
Education								
Bachelor’s degree	154	39.9	4.39 (1.60)	0.702	3.79 (1.93)	< 0.01	39.84 (7.40)	0.07
Master’s degree	182	47.2	4.48 (1.24)		3.48 (1.70)		41.50 (5.24)	
Doctoral degree	50	13.0	4.56 (1.15)		2.82 (1.10)		40.98 (5.92)	
Practice duration (years)								
< 15	266	68.9	4.94 (1.16)	0.272	3.65 (1.88)	0.017	40.76 (6.35)	0.977
≥ 15	120	31.1	4.44 (1.50)		3.23 (1.41)		40.78 (6.22)	
Professional title								
Attending	186	48.2	4.55 (1.38)	0.136	3.70 (1.94)	0.154	40.52 (6.53)	0.582
Associate professor	126	32.6	4.25 (1.54)		3.35 (1.47)		40.76 (6.72)	
Professor	74	19.0	4.55 (1.04)		3.35 (1.68)		41.42 (4.81)	
Participation in CME								
Yes	223	57.8	4.44 (1.38)	0.784	3.67 (1.76)	0.043	40.98 (6.14)	0.448
No	163	42.2	4.48 (1.39)		3.31 (1.73)		40.48 (6.52)	
Annual number of FUO cases								
< 10	70	18.1	4.09 (1.73)	0.042	3.59 (1.99)	0.723	38.87 (8.28)	0.028
≥ 10	316	81.9	4.54 (1.28)		3.50 (1.70)		41.19 (5.70)	
Annual number of MDT for FUO cases								
< 10	222	57.5	4.32 (1.50)	0.02	3.58 (1.84)	0.448	40.14 (7.16)	0.016
≥ 10	164	42.5	4.64 (1.18)		3.44 (1.64)		41.62 (4.79)	
Annual number of academic training for FUO diagnosis and treatment								
< 5	243	63.0	4.29 (1.39)	0.002	3.40 (1.77)	0.073	40.74 (6.51)	0.912
≥ 5	143	37.0	4.73 (1.33)		3.73 (1.72)		40.81 (5.95)	
Prior knowledge of SDM								
Yes	107	27.7	4.88 (1.20)	< 0.01	3.56 ± 1.711	0.768	41.81 (5.33)	0.044
No	279	72.3	4.29 (1.42)		3.5 ± 1.777		40.37 (6.60)	
Prior training on SDM								
Yes	33	8.5	5.24 (1.09)	< 0.01	3.36 ± 1.782	0.598	40.88 (5.49)	0.917
No	353	91.5	4.38 (1.39)		3.53 ± 1.756		40.76 (6.37)	

^a^Total score for attitudes and implementation tendencies regarding the six steps of SDM in daily practice.

^b^Total score for potential barriers to SDM implementation.

^c^Total score on the SDM-Q-Doc, assessing the quality of the medical decision-making process from the specialist’s perspective in a clinical scenario involving bone marrow biopsy for FUO.

CME, continuing medical education; FUO, Fever of Unknown Origin; SDM, Shared decision-making.

### 3.2 Attitudes, potential barriers and implementation

The mean (SD) attitude and implementation tendency score for SDM was 4.46 (1.38; range 0–6). This score was significantly associated with the annual number of FUO cases managed (*p* = 0.042), the annual number of MDT discussions for FUO patients (*p* = 0.020), the amount of FUO-related training received (*p* = 0.002), prior knowledge of SDM (*p* < 0.001), and prior SDM training (*p* < 0.001) (Table 2).

The mean score for potential barriers to SDM implementation was 3.52 (range 0–9, SD 1.76). Higher scores for potential barriers were significantly associated with a lower educational level (*p* < 0.001), a shorter practice duration (*p* = 0.017) and limited participation in CME (*p* = 0.043) (Table 2).

The mean score for the perceived effectiveness of SDM implementation strategies was 40.77 (range 0–45, SD 6.30). Higher scores were significantly associated with the following factors: “gender” (*p* = 0.009), “managing a greater number of FUO cases annually” (*p* = 0.028), “participating in more MDT discussions for FUO each year” (*p* = 0.016), and “prior knowledge of SDM” (*p* = 0.044) (Table 2).

### 3.3 The impact of attitudes and potential barriers on SDM-Q-doc scores

The participants generally exhibited a positive attitude toward the implementation of SDM in the management of patients with FUO. Specifically, the majority of participants (*n* = 359, 93.0%) indicated their willingness to assist patients in evaluating treatment options based on their individual goals and concerns, 331 (85.8%) expressed their readiness to provide information regarding benefits and risks, and 327 (84.7%) indicated their capacity to facilitate deliberation and decision-making. While 236 (61.1%) of participants reported that they would invite patients to participate in decision-making, 246 (63.7%) acknowledged that they did not use collaborative decision-making resources in routine practice. The perceived effectiveness of SDM implementation was significantly associated with five key elements: “inviting patients to participate in SDM” (*p* < 0.001), “presenting the options clearly” (*p* < 0.001), “providing information on benefits and risks” (*p* = 0.002), “assisting patients in evaluating the options based on their goals and concerns” (*p* = 0.006), and “facilitating deliberation and decision-making” (*p* < 0.001) (Table 3).

**Table 3:** Impact of attitudes, implementation tendencies, and potential barriers on sdm implementation.

Variables	Yes/No	*N* = 386	%	Perceived effectiveness^c^	
				Mean (SD)	*P*
Attitude and implementation tendency toward SDM^a^					
Invite patients to participate in SDM	Y	236	61.1	41.82 (4.86)	<0.001
	N	150	38.9	39.12 (7.80)	
Present the options clearly	Y	327	84.7	41.39 (5.25)	<0.001
	N	59	15.3	37.32 (9.70)	
Provide information on benefits and risks	Y	331	85.8	41.39 (5.39)	0.002
	N	55	14.2	37.05 (9.42)	
Assist patients in evaluating the options based on their goals and concerns	Y	359	93.0	41.25 (5.43)	0.006
	N	27	7.0	34.44 (11.72)	
Facilitate deliberation and decision-making	Y	327	84.7	41.47 (5.41)	<0.001
	N	59	15.3	36.90 (9.0)	
Assist with implementation	Y	140	36.3	40.73 (6.01)	0.924
	N	246	63.7	40.79 (6.47)	
Potential barriers to SDM implementation^b^					
Limited time for SDM implementation	Y	161	41.7	40.97 (5.65)	0.599
	N	225	58.3	40.63 (6.73)	
Patients’ unwillingness to participate	Y	100	25.9	39.66 (7.37)	0.068
	N	286	74.1	41.16 (5.85)	
Patients’ disbelief that SDM can improve treatment outcomes	Y	138	35.8	40.03 (6.99)	0.102
	N	248	64.2	41.18 (5.86)	
Uncertainty about the scientific value of SDM	Y	81	21.0	40.63 (5.92)	0.823
	N	305	79.0	40.81 (6.41)	
Doubts regarding the impact of SDM on treatment outcomes	Y	29	7.5	40.97 (5.61)	0.862
	N	357	92.5	40.75 (6.36)	
Insufficient technical support and training for SDM	Y	242	62.7	41.23 (5.49)	0.086
	N	144	37.3	40.00 (7.42)	
Lack of competency and communication skills for SDM	Y	108	28.0	41.16 (5.39)	0.451
	N	278	72.0	40.62 (6.62)	
Patients’ inadequate knowledge	Y	243	63.0	41.39 (5.26)	0.021
	N	143	37.0	39.71 (7.66)	
Unequal information between doctors and patients	Y	256	66.3	41.08 (5.70)	0.213
	N	130	33.7	40.16 (7.32)	

^a^Attitude and implementation tendency toward SDM, assessed by six items corresponding to Questions 12–17 in Table 1.

^b^Potential barriers to SDM implementation, assessed by nine items corresponding to Question 18 in Table 1.

^c^The perceived effectiveness score of SDM implementation strategies.

SDM, shared decision-making.

While the majority of participants (*n* = 357, 92.5%) recognized the benefits of SDM and 305 (79.0%) endorsed its scientific value, several barriers hinder its routine implementation. The three primary barriers identified were as follows: “unequal information between doctors and patients” (*n* = 256, 66.3%), “patients’ inadequate knowledge” (*n* = 243, 63.0%), and “insufficient technical support and training for SDM” (*n* = 242, 62.7%). Furthermore, 161 (41.7%) of participants cited limited time as a barrier, and 138 (35.8%) identified a lack of patient trust as a constraint. Among all potential barriers, only “patients’ inadequate knowledge” was associated with a higher perceived effectiveness of SDM implementation (*p* = 0.021) (Table 3).

### 3.4 Selection of factors influencing SDM-Q-DOC scores

**Figure 2:**
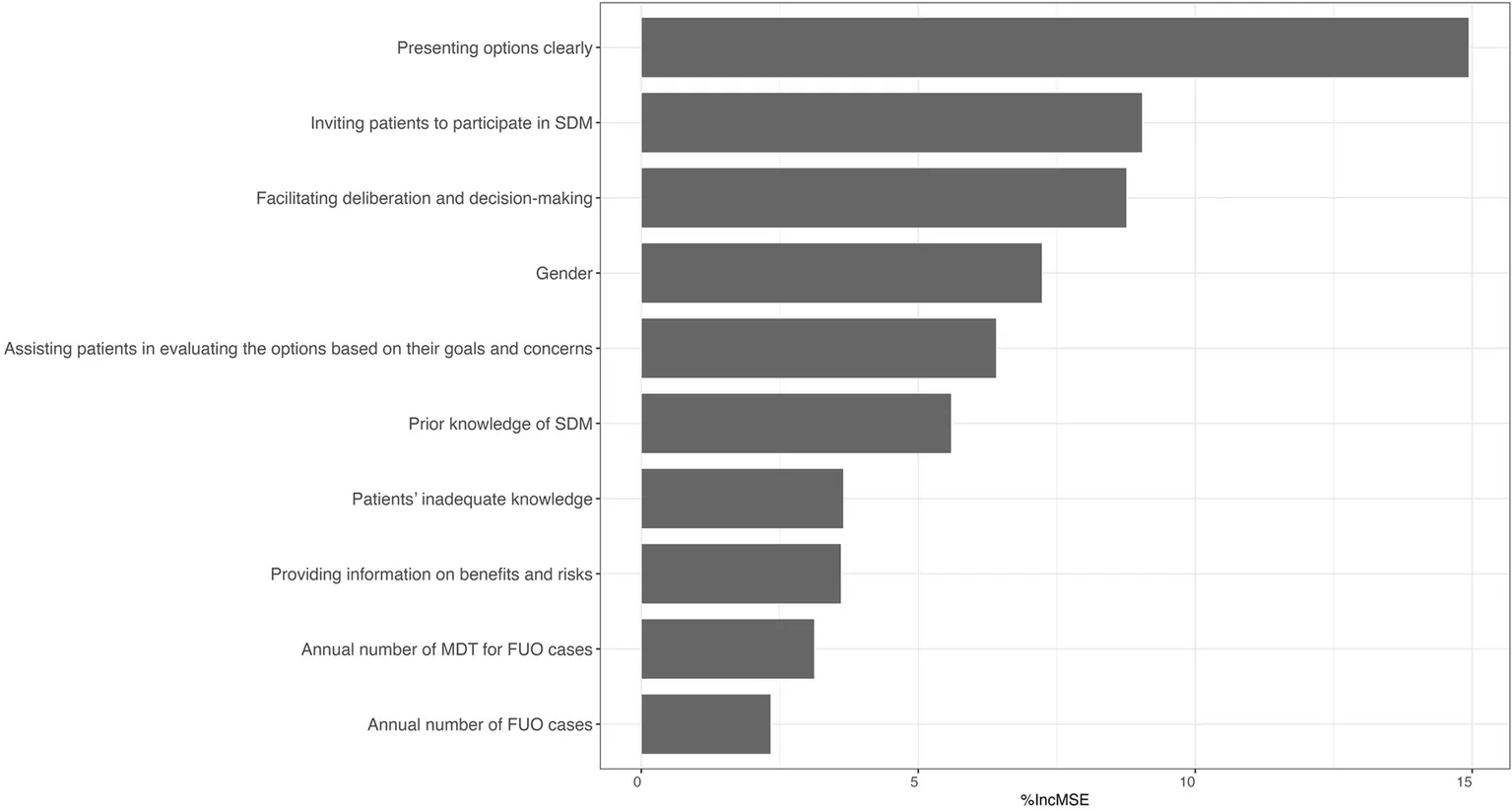
Ranked importance of factors influencing SDM-Q-Doc scores based on the Random Forest model. The variables are ranked by the percentage increase in mean squared error (%IncMSE), where higher values indicate a greater explanatory contribution.

Ten variables that showed statistically significant differences in the univariate analysis were included in a Random Forest model using the SDM-Q-DOC score as the dependent variable. The results indicated that, from highest to lowest, the most influential factors were as follows: “presenting the options clearly,” “inviting patients to participate in SDM,” “facilitating deliberation and decision-making,” “gender,” “assisting patients in evaluating the options based on their goals and concerns,” prior knowledge of SDM, patients’ inadequate knowledge, “providing information on benefits and risks,” annual number of MDT for FUO cases and annual number of FUO cases (Figure 2).

**Figure 3:**
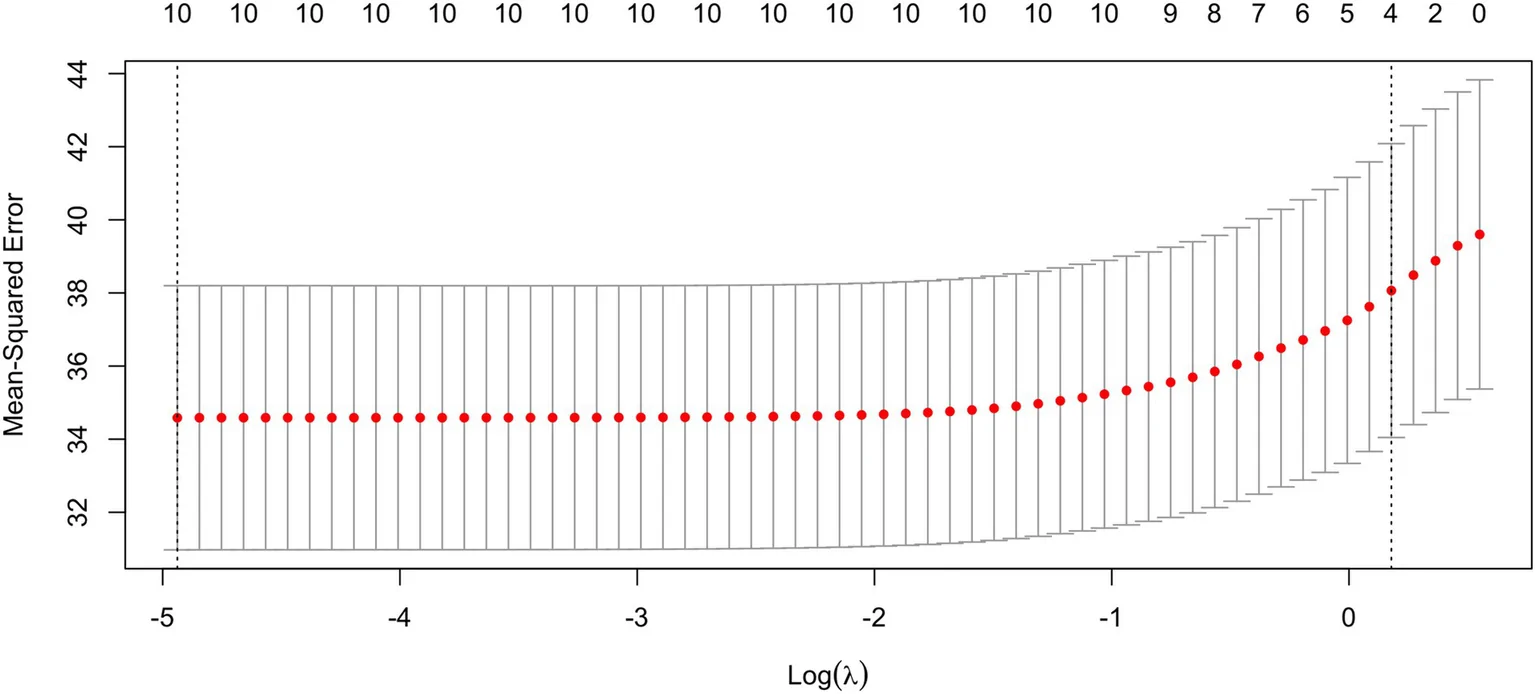
Variable selection via LASSO regression. The figure shows the cross-validation error curves as a function of the log of the regularization parameter (*λ*). The right vertical dashed line indicates the penalty parameter value (λ = 1.196) chosen to select a simplified model containing the four most important variables.

LASSO analysis was performed on the ten statistically significant variables from the univariate analysis, based on the variable importance rankings. The optimal regularization parameter (*λ*) was determined using cross-validation. While both λ_min_ and λ_1se_ are standard selection criteria, the λ_1se_ criterion was adopted (λ = 1.196) to yield a more parsimonious model that maintains robust fit while minimizing complexity. This process resulted in the retention of four key variables (Figure 3).

**Figure 4:**
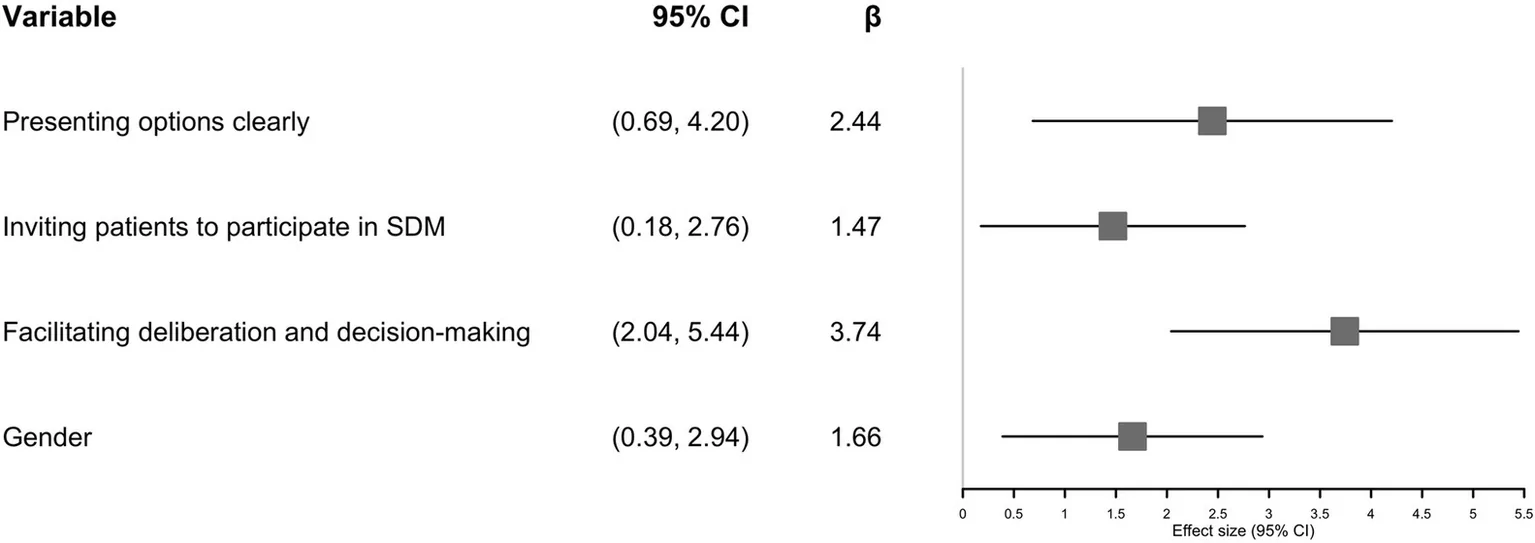
Multivariate linear regression forest plot. The plot presents the effect size (*β*) and 95% confidence intervals (95% CI) for the key predictors identified by the LASSO regression model.

The top four variables, ranked by importance, were therefore identified as key influencing factors and included in the subsequent multiple linear regression analysis. Prior to conducting the multiple linear regression analysis, multicollinearity diagnostics were performed to ensure model stability. The variance inflation factor (VIF) values for all nine independent variables were below 5, indicating no significant multicollinearity. Multiple linear regression analysis was then conducted, and the results indicated that “presenting options clearly” (*p* = 0.007), “inviting patients to participate in SDM” (*p* = 0.026), “facilitating deliberation and decision-making” (*p* < 0.001) and “gender” (*p* = 0.011) were statistically significant predictors of SDM-Q-DOC scores (Figure 4).

## 4 Discussion

SDM has gained increasing attention in clinical practice worldwide. While extensive literature has profoundly advanced our understanding of the clinical, etiological, and diagnostic frameworks of FUO (25, 26), empirical evidence specifically evaluating the SDM process between physicians and patients in this complex scenario remains scarce. This nationwide survey investigated the attitudes, perceived barriers, and effectiveness of SDM implementation strategies for FUO among infectious disease specialists within Chinese general hospitals. To the best of our knowledge, this study offers a novel empirical perspective on SDM implementation in China, providing unique insights to inform future health policy and the evolution of patient-centered practice in high-uncertainty clinical settings.

Our survey revealed that the physicians’ attitudes were significant determinants of the perceived effectiveness of SDM implementation, whereas the perceived barriers did not hinder the implementation. This suggests that interventions aiming to promote SDM may be more effective if they focus on fostering positive attitudes and motivation among physicians, rather than solely addressing perceived obstacles. Given the link between attitudes and SDM behavior, we explored the factors shaping physicians’ attitudes toward SDM further. Physicians with greater clinical experience, prior knowledge of SDM, and participation in SDM training reported a higher perceived effectiveness of SDM implementation. These findings are consistent with previous studies indicating that extensive clinical experience enhances physicians’ communication skills, enabling them to better identify patients’ informational needs and emotional states and advance patient-centered care (27). Furthermore, evidence suggests that targeted SDM training programs can effectively modify healthcare providers’ attitudes and practices, thereby enhancing patients’ willingness to participate in SDM (28, 29). Such training has been shown to strengthen SDM skills in clinical practice by improving communication competencies and promoting active patient engagement (30, 31). For infectious disease specialists, implementing context-specific SDM education is essential for optimising both understanding and the perceived effectiveness of SDM implementation in complex clinical settings. Presently, the primary countries responsible for the development and leadership of most SDM training programs are Canada, the United States, the United Kingdom, and Australia. There are currently no equivalent initiatives in place in China. In consideration of the cultural divergences that are evident among nations, it can be posited that Western training models may not be universally applicable. Consequently, the development of customized SDM training programs, tailored to China’s unique local characteristics, is imperative (32).

Participants identified several potential barriers, including unequal information between doctors and patients (*n* = 256, 66.3%), patients’ inadequate medical knowledge (*n* = 243, 63.0%), and a lack of technical support and training (*n* = 242, 62.7%). These results are broadly consistent with prior research (20, 33–35). Interestingly, inadequate patient knowledge was positively associated with higher SDM-Q-Doc scores. This finding suggests that physicians who recognise this challenge may be more motivated to engage in compensatory communication efforts. Furthermore, physicians with less experience, those with a lower level of educational attainment, and those with limited exposure to CME were found to perceive a greater number of barriers to SDM. It is therefore vital that there is a commitment to ongoing professional development and targeted training to ensure that physicians are equipped with the necessary skills and confidence.

In this study, we incorporated demographic factors, physicians’ attitudes toward SDM, and perceived barriers before implementation as variables. We used Random Forest model and LASSO regression to screen out key predictors, and then included these candidate variables in a multiple linear regression model. From a clinical perspective, this hybrid analytical approach eliminates redundant variables and isolates the most independent and critical predictors of SDM. This approach ensures that our findings are not just mathematically robust, but provide clear, actionable targets for designing future educational interventions and training programs. The results showed that “presenting options clearly,” “inviting patients to participate in SDM” and “facilitating deliberation and decision-making” all had significant positive impacts on the perceived effectiveness of SDM implementation. This finding reinforces the significance of attitude change on behavioral transformation. For instance, Pieterse demonstrated that clear explanation of treatment options and encouraging SDM elevated both decision certainty and satisfaction (36). Elwyn and colleagues conceptualized SDM as involving not only information sharing about options but also supporting patient deliberation (37). Similarly, Shepherd’s “three questions” intervention improved patient engagement and the quality of decision-making (38). Additionally, an interesting finding is that female specialists scored significantly higher in SDM implementation, consistent with previous literature suggesting that female physicians may exhibit greater empathy or communication skills (34, 39). Gender differences in clinical communication have been documented as important determinants of SDM, shaping both clinical practice and the patient-physician relationship (40). For instance, research indicates that male physicians may rely more on heuristics for quicker decisions and take a more instrumental, biomedical approach (41, 42). In contrast, female physicians tend to be more comprehensive in evaluating information (41), while also engaging in more partnership-building and focusing on the psychosocial aspects of health (e.g., emotions, lifestyle, family) (42). These specific behavioral differences are profoundly relevant to the context of adult FUO. The diagnostic landscape of FUO is notoriously complex, rendering rapid heuristics less effective and demanding the comprehensive evaluation style more frequently observed in female physicians. Importantly, as Street notes, these communication patterns should not be linked strictly to gender, but rather to underlying attitudes and teachable skills (42). Healthcare providers’ and patients’ communicative behaviors, attitudes, and perceptions can be effectively modified through well-designed communication skill training. Therefore, these specific strategies must be explicitly integrated into intensive, multi-method SDM training programs for all infectious disease specialists, regardless of gender.

These findings indicate a need to incorporate SDM training into CME programs and clinical guidelines, particularly in specialties dealing with complex, preference-sensitive conditions such as FUO. As an evidence-based approach to patient-centered care, SDM emphasizes equal participation by patients and physicians through bidirectional information exchange and deliberation (8). To effectively promote SDM in clinical practice, it is essential to construct an attitude-focused competency development framework that not only strengthens physicians’ motivation but also equips them with practical skills. Guided by the SDM implementation pathway and informed by best available evidence, this framework should incorporate the design of personalized patient decision aids (PDAs) that provide structured, evidence-based information and support patients in clarifying their values and preferences (38). Training should utilize interactive formats such as simulations, role-playing, video tutorials, case studies, and group discussions (28). Delivered through both online and in-person modalities, these sessions enable physicians to practice using PDAs, improve communication skills, and encourage proactive patient engagement. By combining competency development with tailored decision-support tools, such programs empower physicians to translate positive attitudes into consistent, high-quality SDM behaviors, thereby ensuring treatment decisions align with patients’ values, preferences and goals. Transforming attitudes and behaviors calls for coordinated efforts at all organizational levels. The concept of SDM encompasses a responsibility that extends beyond that of individual physicians, emphasizing the involvement of the entire clinical team. The implementation of this program has been identified as a significant challenge, and as such, calls for comprehensive interventions to support institutions, clinicians, and patients, while ensuring collaboration across different healthcare environments (43). At the institutional level, policies must allocate sufficient time, resources and infrastructure to support this integrated approach in clinical decision-making.

## 5 Limitation

The study has the following limitations that should be considered. Firstly, although this was a nationwide survey, not all provinces in China were represented. Furthermore, the use of convenience sampling via WeChat may introduce potential selection bias, as the participants might not be fully representative of the entire population of infectious disease specialists in China. Stratified sampling could be used in the future to enhance the representativeness and generalizability of the findings. Secondly, it should be noted that all data were self-reported, and therefore subject to recall and social desirability biases. However, a minimum response time threshold was implemented to ensure that participants engaged sufficiently with the survey, thereby improving the precision of the data collected. Lastly, the present study focused mainly on the perspectives of infectious disease specialists and relied on self-reported outcomes. This focus may not fully capture the complexities of shared decision-making from the patient’s standpoint, nor does it provide objective data on clinical recovery or diagnostic accuracy. It is recommended that future research incorporate multi-perspectives from patients and their family members in order to obtain a more comprehensive understanding of the barriers to SDM implementation and the perceived effectiveness of targeted interventions. Longitudinal and interventional studies are also required to assess the sustained impact of SDM on clinical outcomes and comprehensively examine causal relationships among variables.

## 6 Conclusion

Overall, this study demonstrates that infectious disease specialists in Chinese general hospitals exhibited favorable attitudes toward SDM in clinical practice, and these attitudes were significantly associated with enhanced perceived effectiveness of their SDM behaviors. These findings suggest that targeted SDM training programs designed to cultivate positive attitudes may serve as a promising strategy for promoting the adoption and long-term sustainability of SDM in similar clinical settings.

## 7 Practice implications

The findings of this study highlight several promising areas for future research. First, given the demonstrated influence of physician attitudes, future studies should develop and rigorously evaluate the efficacy of culturally adapted, attitude-focused SDM training programs for Chinese physicians. These programs should include the design and validation of PDAs specifically tailored to the Chinese clinical context and complex conditions such as FUO. Second, although this study focused on physician perspectives, it is critical to incorporate the patient’s perspective next. To achieve a patient-centered approach and develop dyadic interventions, it is essential to investigate FUO patients’ preferences, information needs and perceived barriers to SDM. Finally, longitudinal and interventional studies are necessary to establish a causal link between attitude-focused interventions and clinical outcomes. Such research should evaluate the long-term effects of training on physician behavior and SDM adherence, as well as on patient-reported outcomes (e.g., decisional conflict, satisfaction) and clinical indicators in FUO management.

## Data Availability

The raw data supporting the conclusions of this article will be made available by the authors, without undue reservation.
