# Correction: Perspectives of infectious disease specialists on shared decision-making for fever of unknown origin: a national survey in China

**DOI:** 10.3389/fpubh.2026.1978404

**Published:** 2026-09-08

**Authors:** Ling Qin, Zhiyi Pei, Xiaofeng Kang, Taisheng Li, Ying Ge

**Affiliations:** 1 Department of Infectious Diseases, Peking Union Medical College Hospital, Chinese Academy of Medical Sciences & Peking Union Medical College, Beijing, China; 2 School of Nursing, Chinese Academy of Medical Sciences & Peking Union Medical College, Beijing, China; 3 State Key Laboratory of Complex, Severe and Rare Diseases, Peking Union Medical College Hospital, Chinese Academy of Medical Sciences & Peking Union Medical College, Beijing, China

**Keywords:** China, fever of unknown origin, infectious disease specialists, national survey, shared decision-making

The funder [Special Research Fund for the Central High-Level Hospitals of Peking Union Medical College Hospital], [2022-PUMCH-B-043] to [Ying Ge] was erroneously omitted. The corrected **Funding** statement appears below.

“The author(s) declared that financial support was received for this work and/or its publication. This work was supported by the Beijing Educational Science “14th Five-Year Plan” Project (grant number No. 68), Peking Union Medical College Young Teachers Program (grant number 2025mesp002), and Special Research Fund for the Central High-Level Hospitals of Peking Union Medical College Hospital (grant number 2022-PUMCH-B-043).”

The original version of this article has been updated.

In the original publication, affiliations 4 and 5 were erroneously included for author Taisheng Li. These inaccurate affiliations have been removed.

The original version of this article has been updated.

